# Is the production of reactive oxygen and nitrogen species by macrophages associated with better infectious control in female mice with experimentally disseminated and pulmonary mucormycosis?

**DOI:** 10.1371/journal.pone.0270071

**Published:** 2022-12-15

**Authors:** Amanda Ribeiro dos Santos, Thais Fernanda Fraga-Silva, Débora de Fátima Almeida-Donanzam, Angela Carolina Finatto, Camila Marchetti, Maria Izilda Andrade, Olavo Speranza de Arruda, Maria Sueli Parreira de Arruda, James Venturini

**Affiliations:** 1 Faculdade de Medicina, Universidade Federal de Mato Grosso do Sul (UFMS), Campo Grande, Mato Grosso do Sul, Brazil; 2 Departamento de Bioquímica e Imunologia, Escola de Medicina de Ribeirão Preto, Universidade de São Paulo, São Paulo, São Paulo, Brazil; 3 Faculdade de Ciências, Universidade Estadual Paulista (Unesp), Bauru, São Paulo, Brazil; 4 Lauro de Souza Lima Institute, Bauru, São Paulo, Brazil; University of Michigan Health System, UNITED STATES

## Abstract

Different levels of resistance against *Rhizopus oryzae* infection have been observed between inbred (BALB/c) and outbred (Swiss) mice and are associated with the genetic background of each mouse strain. Considering that macrophages play an important role in host resistance to *Rhizopus* species, we used different infectious outcomes observed in experimental mucormycosis to identify the most efficient macrophage response pattern against *R*. *oryzae in vitro* and *in vivo*. For this, we compared BALB/c and Swiss macrophage activity before and after intravenous or intratracheal *R*. *oryzae* infections. The production of hydrogen peroxide (H_2_O_2_) and nitric oxide (NO) was determined in cultures of peritoneal (PMΦ) or alveolar macrophages (AMΦ) challenged with heat-killed spores of *R*. *oryzae*. The levels of tumor necrosis factor-alpha (TNF-α) and interleukin-10 (IL-10) were measured to confirm our findings. Naïve PMΦ from female BALB/c mice showed increased production of H_2_O_2_, TNF-α, and IL-10 in the presence of heat-killed spores of *R*. *oryzae*. Naïve PMΦ from female Swiss mice were less responsive. Naïve AMΦ from the two strains of female mice were less reactive to heat-killed spores of *R*. *oryzae* than PMΦ. After 30 days of *R*. *oryzae* intravenous infection, lower fungal load in spleen from BALB/c mice was accompanied by higher production of H_2_O_2_ by PMΦ compared with Swiss mice. In contrast, AMΦ from BALB/c mice showed higher production of NO, TNF-α, and IL-10 after 7 days of intratracheal infection. The collective findings reveal that, independent of the female mouse strain, PMΦ is more reactive against *R*. *oryzae* upon first contact than AMΦ. In addition, increased PMΦ production of H_2_O_2_ at the end of disseminated infection is accompanied by better fungal clearance in resistant (BALB/c) mice. Our findings further the understanding of the parasite–host relationship in mucormycosis.

## 1. Introduction

Mucormycosis is a devastating invasive fungal infection predominantly caused by *Rhizopus* species [1]. It has emerged as a global public health threat during the COVID-19 pandemic [2–4]. Infection occurs via diverse routes, such as inhalation, percutaneous, or ingestion of spores. Mucormycosis can have a wide variety of manifestations, with rhinocerebral and pulmonary diseases being the most common [5]. The mortality rate varies from 46% to 70%, and is up to 90% upon dissemination [6–8].

Risk factors for mucormycosis include diabetes mellitus, neutropenia, sustained immunosuppressive therapy, chronic prednisone use, and iron chelation therapy [7]. During the COVID-19 pandemic, the use of immunomodulatory drugs (e.g., systemic corticosteroids and tocilizumab) and COVID-19-induced immune dysregulation have increased the risk of mucormycosis [9, 10]. Cases of COVID-19 associated mucormycosis (CAM) have been reported worldwide [11–20]. CAM cases have mostly been associated with underlying uncontrolled diabetes mellitus-related diseases. Cutaneous and soft-tissue mucormycosis have been observed in immunocompetent individuals [21, 22], such as those induced by traumatic implantation of contaminated soil and water during tornadoes [23].

Considering the high rates of mortality and morbidity associated with this life-threatening disease [14], the prognosis and outcome of mucormycosis have not improved significantly improved over the last decades, mainly because of the difficulty in early diagnosis and the limited activity of current antifungal agents against Mucorales [24, 25]. In addition, the pathogenesis of mucormycosis remains incompletely understood, particularly in relation to the parasite–host relationship.

Experimental models of mucormycosis are widely used for the evaluation of antifungal therapy. In these studies, immunosuppression is used to induce fungal dissemination [26–29]. Nevertheless, the effects of immunosuppressive drugs restrict the evaluation of the immunological mechanisms involved in fungal resistance. Recently, our group has focused on the *Rhyzopus*–host interplay using immunocompetent models, inducing both disseminated and pulmonary mucormycosis [30, 31]. Using BALB/c and Swiss mice, we determined resistant and less resistant models of mucormycosis. BALB/c mice have a better capacity for decreased fungal load during 30 days of intravenous and intratracheal infection. However, Swiss mice are less responsive to *R*. *oryzae* infection and consequently have more prolonged viable fungal presence in internal organs [30, 31]. We and others [32, 33] have highlighted the importance of evaluating the immune response in immunocompetent mice to understand the mechanisms involved in the resistance to Mucorales agents, as well as the pathogenesis of mucormycosis in immunocompetent individuals [21, 22].

Andrianaki et al. revealed the essential role of *Rhizopus*–macrophage interplay in pulmonary mucormycosis. The authors used an immunocompetent model to demonstrate that the development of mucormycosis is related to prolonged intracellular survival of the fungus inside macrophages [33]. Considering the poor knowledge of macrophage activity during mucormycosis in the context of natural resistance, in the present study, we employed resistant and less resistant mouse models to determine reactive oxygen and nitrogen species production by *R*. *oryzae*-infected macrophages.

## 2. Material and methods

### 2.1 Mice

Two-month-old female inbred BALB/c and outbred Swiss mice from the Animal House at the Laboratório de Imunopatologia Experimental of University Estadual Paulista, Bauru, Brazil (UNESP) were randomly divided into groups. Food and water were provided *ad libitum*. This study was conducted in strict accordance with the recommendations of the Guide for the Care and Use of Laboratory Animals of the National Institutes of Health and the Brazilian College of Animal Experimentation. The study was approved by the Institutional Animal Care and Use Committee (Protocol Number:1608/46/01/2013-CEUA-FC) of the Animal Experimentation Ethics Committee of the School of Sciences of Bauru, UNESP. All surgeries were performed under ketamine/xylazine anesthesia. Every effort was made to minimize suffering.

### 2.2 Fungal strains

*R*. *oryzae* (IAL 3796) was previously obtained from the fungal collection of the Instituto Lauro de Souza Lima. Species identification was performed at the Adolfo Lutz Institute (São Paulo, Brazil). The fungi were maintained by monthly subculturing on Sabouraud dextrose agar slants (Difco Laboratories, Detroit, MI, USA).

### 2.3 Experimental design

The mice were randomly separated into two main groups according to the route of inoculation: intravenous (*Rhi-*IV*)* and intratracheal (Rhi-IT) groups. For the *Rhi-*IV group, Swiss and BALB/c mice were inoculated with 3.0 × 10^4^ viable spores of *R*. *oryzae* in the caudal vein. For the *Rhi-*IT groups, Swiss and BALB/c mice were inoculated with 2.0 × 10^6^ viable spores of *R*. *oryzae* in the trachea. Next, groups of six *R*. *oryzae*-infected mice were evaluated on days 7 and 30 post-infection. The non-infected groups comprised BALB/c and Swiss mice inoculated with sterile saline solution (SSS) via intravenous or intratracheal routes.

### 2.4 Fungal infection

The fungi were washed carefully with SSS, and the suspension was mixed twice for 10 s on a vortex mixer and decanted for 5 min. Supernatants were collected and washed twice. We determined fungal viability using cotton blue staining. In the *Rhi*-IV group, 100 μL of fungal suspension (3 × 10^4^ spores of *R*. *oryzae*) was inoculated into the lateral tail vein. In the *Rhi-*IT group, mice were anesthetized via intraperitoneal administration of ketamine and xylazine at doses of 80 and 10 mg/kg body weight, respectively. After tracheal exposition, each mouse was inoculated with 2 × 10^6^ Rhizopus oryzae spores in 40-μL of the suspension. The incision was sutured with surgical thread, and the animals were kept in a warm place and observed for recovery.

### 2.5 Collection of biological material

The mice were anesthetized with isoflurane and euthanized via CO_2_ asphyxiation. Peritoneal lavage (PL) and bronchoalveolar lavage (BAL) were performed in the *Rhi*-IV and *Rhi-*IT groups, respectively, using cold and sterile phosphate-buffered saline. Fragments of the brain, liver, lungs, spleen, and kidneys were collected and subjected to microbiological evaluation.

### 2.6 Recovery of viable fungi

Quantitative colony culture is a widely used method for organ fungal burden determination per gram of tissue. Typically, whole tissue is ground to form a suspension before inoculation onto culture plates. However, this method does not preserve viability of Mucorales [21]. To avoid damage to fragile hyphae and consequent false-negative results in our quantitative analysis [34], we used the fragment method in our set of experiments, as previously reported [30, 31, 35]. Ten fragments (2 × 2 mm) of brain, liver, lung, spleen, and kidney tissues were cultured on Sabouraud agar plates at 25°C for a maximum of 7 days. Fungal growth from the fragments was enumerated and expressed as the frequency of *R*. *oryzae*-positive fragments in the total cultivated area.

### 2.7 Macrophage culture

BAL and PL suspensions were centrifuged for 10 min at 410 *× g*. The cells were resuspended in 1.0 mL of RPMI-1640 (Nutricell, Campinas, Brazil) supplemented with 10% heat-inactivated fetal calf serum (Nutricell), penicillin (100 UI mL^-1^), streptomycin (100 mg mL^-1^; Sigma-Aldrich, St. Louis, MO, USA), and amphotericin B (0.25 μg mL^-1^; (Sigma-Aldrich). The cell concentration was adjusted to 1.0 × 10^5^ mononuclear phagocytes mL^-1^, as judged by the uptake of 0.02% neutral red (Sigma-Aldrich) and confirmed by the expression of F4/80 by fluorescence-activated cell sorting. Cells were plated in 96-well flat-bottomed microtiter plates (Greiner BioOne, Frickenhausen, Germany) and incubated for 2 h at 37°C in a 5% CO_2_ atmosphere in a humidified chamber to allow cells to adhere and spread. Non-adherent cells were removed by washing the wells three times with RPMI. The remaining adherent cells, which comprised > 95% mononuclear phagocytes as assessed by morphological examination, were used for the experiments. The adherent cells were cultured at 37°C and 5% CO_2_ in supplemented RPMI-1640 with or without heat-killed spores of *R*. *oryzae* (*R*. *oryzae*-Ag) at a spore:cell ratio of 1:1. As an internal control for macrophage activity, the cells were cultured with 10 μg ml-1 lipopolysaccharide (Sigma-Aldrich). After 24 h, cell-free supernatants were harvested and stored at -80°C for cytokine analysis.

### 2.8 Production of hydrogen peroxide (H_2_O_2_)

The production of H_2_O_2_ was estimated as described by Russo et al. [36]. Briefly, adhered mononuclear phagocytes obtained as previously described were maintained in RPMI-1640 culture medium at 37°C and 5% CO_2_ for 24 h. At the end of the cell culture period, the supernatants were removed from the wells. Wells received a phenol red solution containing dextrose (Sigma-Aldrich), phenol red (Sigma-Aldrich), and horseradish peroxidase type II (Sigma-Aldrich). Plates were incubated at 37°C in 5% CO_2_ for 1 h. The reaction was stopped by adding 1 N NaOH. H_2_O_2_ concentration was determined using an ELx 800 colorimetric microplate reader (BioTek Instruments Inc., Winooski, VT, USA) as previously described [37].

### 2.9 Detection of levels of nitric oxide (NO)

NO production was estimated using the Griess method as described by Green et al. [38]. The production of nitrite, a stable end product of NO, was measured in cell-free supernatants of cultured adhered mononuclear phagocytes. Briefly, a cell-free volume of 0.1 mL was incubated with an equal volume of Griess reagent for 10 min at 25–27°C. Griess reagent was prepared using1% sulfanilamide (Synth, Diadema, Brazil), 0.1% naphthalene diamine dihydrochloride (Sigma-Aldrich), and 2.5% H_3_PO_4_. Nitrite accumulation was quantified using the aforementioned ELx 800 colorimetric microplate reader (BioTek Instruments). The nitrite concentration was determined using sodium nitrite (Sigma-Aldrich) diluted in RPMI-1640 medium as a standard [37].

### 2.10 Dosage of tumor necrosis factor-alpha (TNF-α) and interleukin-10 (IL-10)

TNF-α and IL-10 levels were measured in cell-free supernatants of cell cultures using a cytokine Duo-Set Kit (R&D Systems, Minneapolis, MN, USA), according to the manufacturer’s instructions.

### 2.11 Statistical analyses

Normality tests of data were performed using the Shapiro–Wilk’s test. To compare two independent samples, the *t*-test was performed. For multiple comparisons, analysis of variance (ANOVA) with Tukey’s post-hoc test was used. Pearson’s correlation coefficient was used to measure the statistical association between two continuous variables. All statistical analyses were performed using GraphPad Prism version 5.0 for Windows (GraphPad Software, San Diego, CA, USA). A p-value ≤5% was considered statistically significant.

## 3. Results

### 3.1 Production of reactive oxygen reactive species and IL-10 by heat-killed *R*. *oryzae*-infected peritoneal macrophages is dependent on mouse genetic background

First, we evaluated the *in vitro* responses of peritoneal (PMΦ) and alveolar (AMΦ) macrophages obtained from non-infected BALB/c and Swiss mice challenged with heat-killed *R*. *oryzae* (*R*. *oryzae*-Ag). To evaluate the PMΦ and AMΦ responses, we measured the reactive oxygen and nitrogen species (H_2_O_2_ and NO) in the cultures. We also measured TNF-α and IL-10 levels to validate our findings.

**Fig 1:**
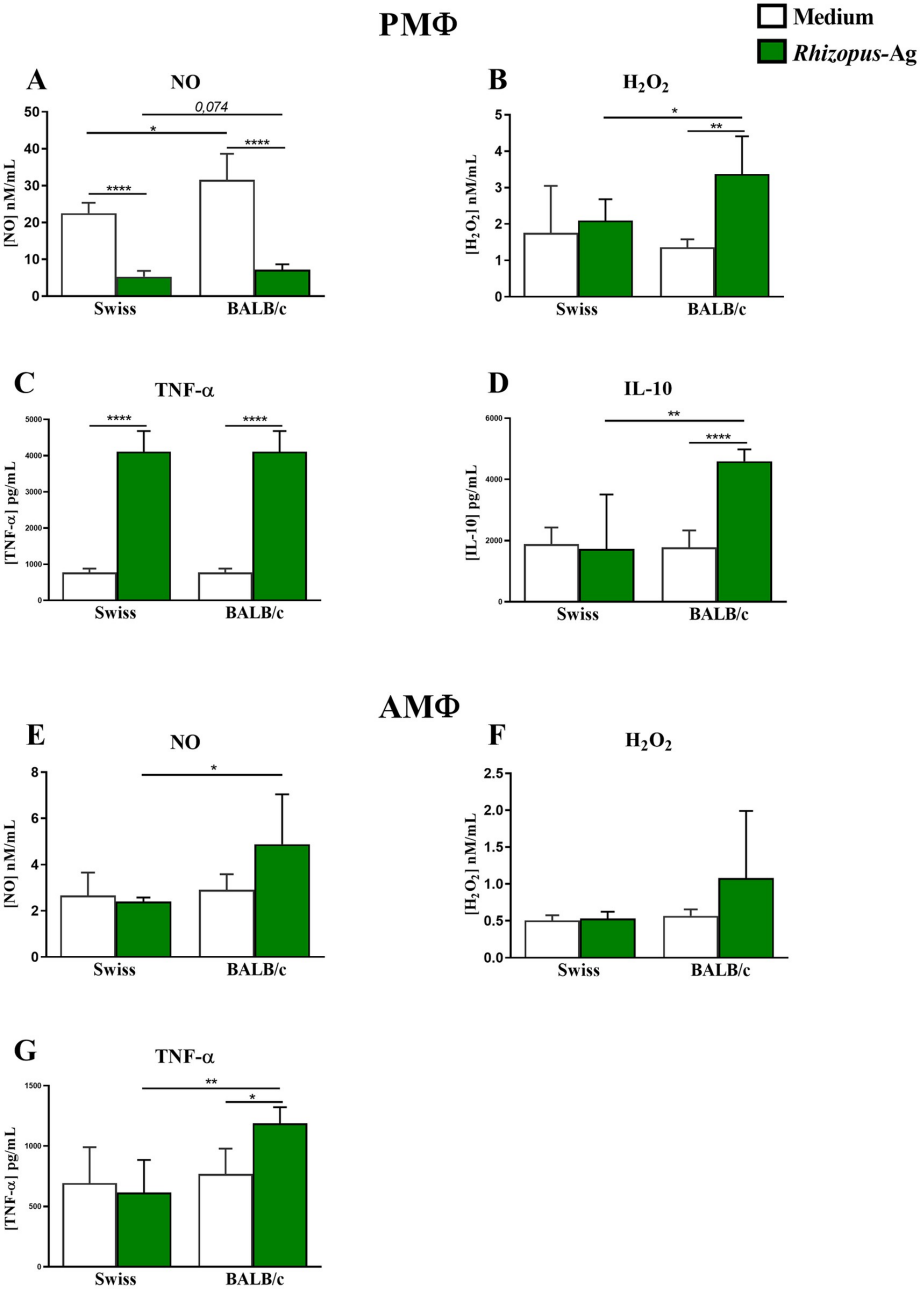
Peritoneal and alveolar macrophages from naïve Swiss and BALB/c mice react differently to heat-killed *R*. *oryzae*. (A) Nitric oxide (NO), (B) hydrogen peroxide (H_2_O_2_), (C) tumor necrosis factor-alpha (TNF-α), and (D) interleukin 10 (IL-10) levels in cell-free supernatants of peritoneal macrophages (PMΦ). (E) NO (F) H_2_O_2_, and (G) TNF-α levels in cell-free supernatants of alveolar macrophages (AMΦ) of non-infected Swiss, and BALB/c mice co-cultured or not co-cultured with heat-killed spores of *R*. *oryzae*. Student’s t-test; n = 5–7; *p < 0.05, **p< 0.01, ***p< 0.001.

In the presence of heat-killed spores of *R*. *oryzae*, PMΦ from the two mouse strains showed decreased NO production (Fig 1A) and increased TNF-α production (Fig 1C). However, PMΦ from the BALB/c strain showed higher NO production than PMΦ from Swiss mice under the same culture conditions (Fig 1A). Interestingly, *R*. *oryzae*-infected PMΦ from BALB/c mice showed increased production of H_2_O_2_ and IL-10 (Fig 1B and 1D). Additionally, PMΦ from the BALB/c strain showed higher production of H_2_O_2_ and IL-10 in the presence of *R*. *oryzae*-Ag when compared with antigen-stimulated PMΦ from Swiss mice (Fig 1B and 1D).

In contrast to PMΦ, AMΦ from both Swiss and BALB/c mice did not show differences in the production of both NO and H_2_O_2_ in the presence of *R*. *oryzae*-Ag (Fig 1E and 1F). Similar to PMΦ, AMΦ from BALB/c mice showed increased TNF-α production in the presence of *R*. *oryzae*-Ag (Fig 1G). Additionally, AMΦ from BALB/c mice also showed higher production of NO and TNF-α in the presence of *R*. *oryzae*-Ag compared with antigen-stimulated AMΦ from Swiss mice (Fig 1E and 1G). In this set of experiments, no detectable IL-10 production was observed.

### 3.2 Enhanced production of H_2_O_2_ by PMΦ is associated with better clearance of *R*. *oryzae* in a model of disseminated mucormycosis

**Fig 2:**
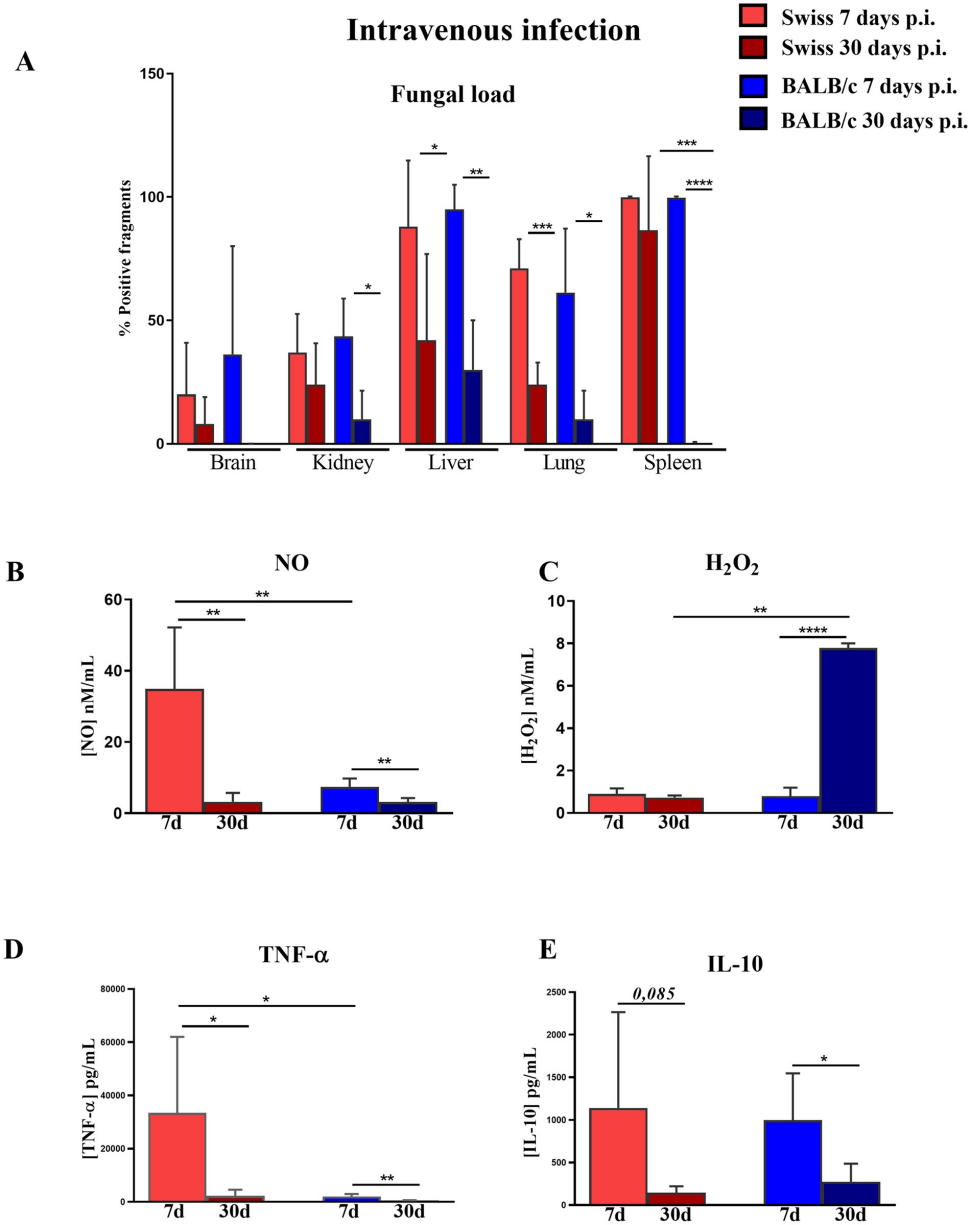
Increased PMΦ production of H_2_O_2_ by peritoneal macrophages (PMΦ) at the end of disseminated infection is related to efficient fungal clearance in BALB/c mice. (A) Total fungal load (B) Nitric oxide (NO), (C) hydrogen peroxide (H_2_O_2_), (D) tumor necrosis factor-alpha (TNF-α), and (E) interleukin-10 (IL-10) levels in cell-free supernatants of PMΦ from Swiss or BALB/c mice co-cultured with heat-killed spores of *R*. *oryzae*. Linear regression analysis between H_2_O_2_ levels and total fungal load in BALB/c (F) and Swiss (G) mice. The infected group was composed of mice inoculated intravenously with 3 × 10^4^ spores of *R*. *oryzae* and evaluated after 7 and 30 days. Any significant differences relative to infected samples compared to different times post-infection (letters) and different strains (*) are indicated. Student’s t-test; n = 5–7; *p < 0.05, **p< 0.01, ***p< 0.001.

In the present study, BALB/c and Swiss mouse strains generally showed more and less resistance, respectively, against *in vivo R*. *oryzae* infection. Seven days following intravenous infection, both strains of mice showed viable fungal recovery in the brain, kidney, liver, lungs, and spleen. As previously reported (30,31), the capacity to decrease fungal load in different organs was different among *Swiss* and BALB/c mice. While Swiss mice showed a reduction in the fungal load only on the liver and lungs after 30 days of infection, BALB/c mice showed a significant decrease in the fungal load on the kidney, liver, lung, and spleen at the same time post-infection. When the fungal load was compared between different strains of mice, we observed that at 30 days following infection BALB/c mice had a lower fungal load in the spleen than Swiss mice. No differences in the fungal load in the brain, kidney, liver, or lung were observed between the two strains of mice (Fig 2A).

Considering the role of the spleen in the immunological response against intravenous infections and to explain the results of *in vitro R*. *oryzae-*infected macrophage experiments, we hypothesized that macrophage activity represented by the production of reactive species of oxygen and nitrogen by macrophages could be involved in the spleen differential response observed between these two mouse strains.

To test our hypothesis, we first evaluated the specific activity of PMΦ derived from BALB/c and Swiss mice intravenously infected with *R*. *oryzae*. On days 7 and 30 p.i., PMΦ were recovered from the peritoneal cavity and challenged with heat-killed spores of *Rhizopus oryzae*.

On day 7, PMΦ from Swiss mice showed a higher production of NO (Fig 2B) and TNF-α (Fig 2D) than PMΦ from BALB/c mice. However, PMΦ from both strains of mice displayed decreased levels of NO (Fig 2B), TNF-α (Fig 2D), and IL-10 (Fig 2E) after 30 days of infection compared to the initial days of infection (7 days post-infection). In contrast, on day 30, more resistant BALB/c mice showed higher levels of H_2_O_2_ (Fig 2C). The production of IL-10 did not differ between the two mouse strains (Fig 2E).

### 3.3 Enhanced initial pro-inflammatory response by AMΦ of mice seems to better control pulmonary mucormycosis

**Fig 3:**
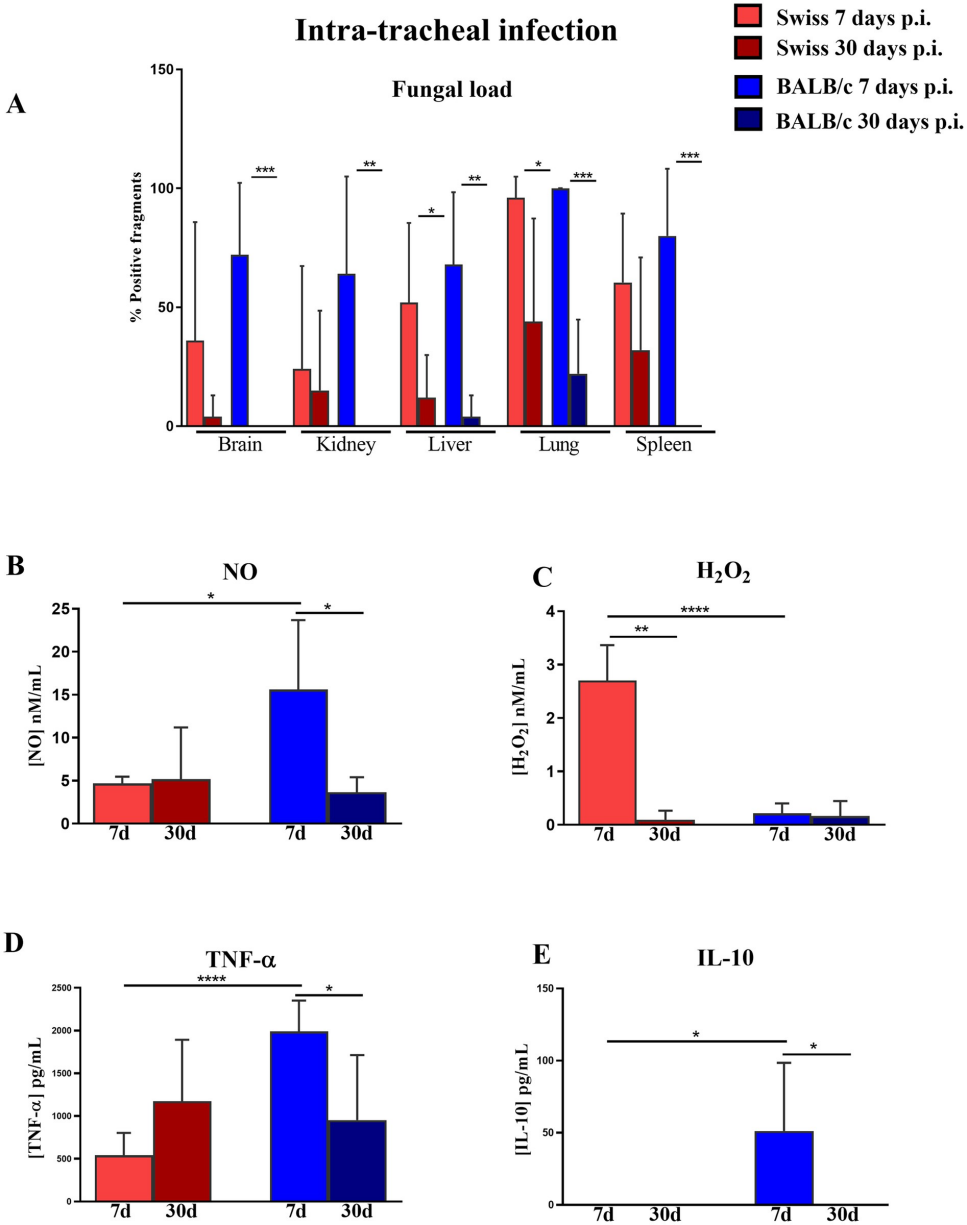
AMΦ from BALB/c mice display higher production of nitric oxide (NO), tumor necrosis factor-alpha (TNF-α), and interleukin-10 (IL-10) than AMΦ from Swiss mice after 7 days of *R*. *oryzae* pulmonary infection. (A) Total fungal load. (B) NO, (C) H_2_O_2,_ (D) TNF-α, and (E) IL-10 levels in cell-free supernatants of AMΦ from Swiss or BALB/c mice co-cultured with heat-killed spores of *R*. *oryzae*. The infected group comprised mice inoculated intratracheally with 2 ×10^6^ spores of *R*. *oryzae* and evaluated after 7 and 30 days. Significant differences relative to infected samples compared to different times post-infection (letters) and to different strains (*) are indicated. Student´s t-test; n = 5–7; *p < 0.05, **p< 0.01, ***p< 0.001.

To evaluate tissue-specific responses, BALB/c and Swiss mice were intratracheally infected with *R*. *oryzae*. As observed with intravenous infection, BALB/c mice showed better fungal clearance than Swiss mice (Fig 3A). Similar to intravenous infection, after 7 days of intratracheal infection, both strains of mice showed viable fungal recovery in the brain, kidney, liver, lungs, and spleen. Swiss mice also showed a reduction in the fungal load only in the liver and lungs after 30 days of infection, whereas BALB/c mice showed significantly decreased fungal load in all evaluated organs (Fig 3A). However, we did not observe differences in the fungal load on the brain, kidney, liver, and lungs between the two strains of mice (Fig 3A).

Considering that both strains of mice were effective in decreasing the fungal load of the lungs and we did not observe differences in viable fungal recovery in this organ between the two strains of mice, we tried to identify a pattern of AM response for both strains of mice that could be related to an efficient response for in vivo infection. In contrast to what was observed with intravenous infection, we observed that after 7 days of intratracheal infection, AMΦ from BALB/c mice showed higher production of NO (Fig 3B), TNF-α (Fig 3D), and IL-10 (Fig 3E) than AMΦ from Swiss mice. In contrast, AM from Swiss mice showed higher production of H_2_O_2_ than AM from BALB/c mice (Fig 3C). After 30 days of infection, the production of H_2_O_2_ decreased in Swiss mice, as did the production of NO (Fig 3B), TNF-α (Fig 3D), and IL-10 (Fig 3E). Additionally, on day 30, no differences were observed in the production of NO, H_2_O_2_, TNF-α, and IL-10 by AMΦ (Fig 3B–3E).

## 4. Discussion

In the present study, the different outcomes observed in experimental mucormycosis between immunocompetent female BALB/c and Swiss mice were used to highlight the protective pattern of macrophage responses against *R*. *oryzae*. Macrophages from different microenvironments showed different responses to *R*. *oryzae in vitro* and during *in vivo* infection. In addition, a better outcome was accompanied by higher H_2_O_2_ production by PM during experimental disseminated mucormycosis. In addition, our observations indicate that the genetic background interferes with the macrophage response.

Inbred mouse strains vary widely in their degree of innate susceptibility to systemic fungal infections [39–43]. BALB/c and Swiss mice are resistant and susceptible strains, respectively, to experimental infections caused by *Cryptococcus neoformans* [41], *Candida albicans* [40, 44], and *Paracoccidioides brasiliensis* [43]. The genetic target C5-deficiency (Hc0 allele, hemolytic complement) modulates the host’s initial response and causes susceptibility and ineffective inflammatory response against fungal infections [40, 41]. C5 is a powerful chemotactic factor for polymorphonuclear leukocytes that is active as an anaphylatoxin. The lack of C5 blocks complement, thereby decreasing the opsonization and recruitment of phagocytic cells [41]. Although the higher susceptibility of Swiss mice to fungal infections may be explained by a deficiency in the Hc0 allele [43–45], our results suggest that other genetic targets may be involved in the generally lower responsiveness of macrophages from Swiss mice to heat-killed *R*. *oryzae*.

According to clinical and experimental data, individuals who lack phagocytes or have impaired phagocytic functions have a higher risk of developing mucormycosis [5, 6, 46]. The present *in vitro* observations indicated that peritoneal macrophages from the most resistant strain (BALB/c) had increased production of inflammatory mediators, such as H_2_O_2_ and TNF-α [47], in the first contact with *R*. *oryzae* antigen. A previous *in vitro* study showed that inactivated Mucorales cells can stimulate high levels of TNF-α, IL-1β, IL-6, IL-8, granulocyte macrophage colony-stimulating factor, and monocyte chemoattractant protein-1 production by human cells of different immune subsets, with parallel upregulation of the transcriptional activity of IL-1β and TNF-α [48, 49]. As oxidative microbicidal production by neutrophils and monocytes is related to *R*. *oryzae* hyphae damage and killing [50, 51], our results indicate that an initial pro-inflammatory response against *R*. *oryzae* can led to a better outcome in disseminated experimental mucormycosis. More experiments are needed to explore the role of PMΦ response in the control of mucormycosis disseminated infection.

Unexpectedly, in the first contact with *R*. *oryzae* antigen, PMΦ, in general, decreased the production of NO. Similar to our results, it was demonstrated in another study that *Aspergillus nidulans* melanin inhibits the NO production by lipopolysaccharide (LPS)-stimulated PMΦ accompanied by a slight stimulatory effect on TNF-α production [52]. Melanin is a pigment in the fungal cell wall of black molds that has been shown to block the effects of hydrolytic enzymes on the cell wall [53]. Regardless of this topic and considering the observations made in this study, the immunomodulatory effects of the *R*. *oryzae* cell wall on PMΦ need to be further investigated.

In contrast to PMΦ, AM for both strains of mice was less reactive against heat-killed *R*. *oryzae*-ag. AMΦ are the most abundant antigen-presenting cells in the lungs, and they play a critical role in regulating pulmonary immune responses to inhaled pathogens and allergens. However, compared to other macrophages, AMΦ are phenotypically different [54]. Most studies indicate that AMΦ are less inflammatory and are ineffective at initiating immune responses relative to other antigen-presenting cells in the lung, which may serve to limit deleterious inflammatory responses within the lungs [55]. Even so, AMΦ from BALB/c mice were more reactive in the first contact with heat-killed *R*. *oryzae*, showing higher production of NO and TNF-α than naïve AMΦ from Swiss mice. This pattern was also observed during the in vivo infection response.

In general, the present in vivo data showed that after 30 days of *R*. *oryzae* intravenous infection, the fungal load in the spleen was lower in BALB/c mice than that in Swiss mice, accompanied by a more remarkable difference in activity of PMΦ at this point of infection. However, after 30 days of *R*. *oryzae* pulmonary infection, no significant differences in lung fungal load between the two strains of mice, accompanied by no differences in alveolar macrophage activity at this point of infection.

After pulmonary infection, AMΦ from the BALB/c strain produced higher levels of NO, TNF-α, and IL-10 than those in Swiss mice. In contrast, AMΦ from Swiss mice produced higher levels of H_2_O_2_ than AMΦ from BALB/c mice. In addition to the differences in the pattern of response of AMΦ between Swiss and BALB/c mice, both were able to significantly decrease the recovery of viable fungal load in the lungs after 30 days of infection. Pulmonary activated macrophages are a major defense against fungal invasion [56]. In contact with macrophage receptors, TNF-α provides signals that lead to induction of antimicrobial activity. This activity is dependent on NO synthase activation and production [57]. Some studies have demonstrated that this process is essential for fungal cell death. In paracoccidioidomycosis, TNF-α induces *P*. *brasiliensis* killing by H_2_O_2_ and NO release [58]. In cryptococcosis, TNF-α significantly promotes macrophage NO production and anti-cryptococcal activity [59]. In addition, TNF-α enhances pulmonary alveolar macrophage phagocytosis and oxygen production during initial contact with *A*. *fumigatus* [60]. TNF-α contribute to the influx and activation of neutrophils and mononuclear cells in the lungs during filamentous fungal challenges [61]. In mucormycosis, TNFα signaling may have a protective response, since patients treated with a tumor necrosis factor inhibitor have a higher risk of developing disseminated mucormycosis [62, 63]. In addition to the lack of knowledge about the immune response in the lung during pulmonary mucormycosis, our results suggest that an initial inflammatory response of AMΦ mediated by NO and TNF-α and/or H_2_O_2_ seems to be important for infection control.

Although the association of NO with the killing of yeast cells during *in vitro* experiments supports the importance of this metabolite in host protection [64], other studies have demonstrated that the overproduction of this metabolite is associated with susceptibility to experimental PCM [65, 66]. Nascimento et al. [66] observed that high levels of NO induce T-cell immunosuppression during *P*. *brasiliensis* infection. These reports suggest that the protective or deleterious role of NO *in vivo* depends on the balance between fungicidal and immunosuppressive properties.

IL-10 is an anti-inflammatory cytokine that impedes pathogen clearance. However, it can also ameliorate immunopathology [67]. The balance between pro-and anti-inflammatory cytokines is crucial for host defense against *A*. *fumigatus*. An *in vitro* study of mononuclear cells stimulated with *A*. *fumigatus* showed that hyphae of this fungus induced the release of IL-10 by mononuclear cells. The process was dependent on endogenous IL-1 [68]. Inflammatory cytokines, such as IFN-γ, TNF-α, and IL-18, activate monocytes and neutrophils to ingest and kill *A*. *fumigatus* conidia and hyphae, and the subsequent release of the anti-inflammatory cytokine IL-10 is responsible for downregulating the potential deleterious overstimulation induced by inflammatory mediators [69].

In general, one of the biological effects of IL-1 is increased synthesis of IL-10 [70]. Netea et al. [71] showed that *Candida* can stimulate release of IL-1 and IL-10 by cells [71]. In mucormycosis, although high production of IL-10 by mucorales-specific T cells from patients has been related to susceptibility in the late phase of the disease [72], we observed a correlation between higher IL-10 production by alveolar macrophages in the initial stages of *R*. *oryzae* infection and better outcomes in experimental pulmonary mucormycosis. We suggest that high levels of IL-10 have a biological effect on balancing the pro-inflammatory response mediated by high levels of TNF-α and NO. Fundamental step in an efficient immune response.

While AMΦ from a more resistant strain showed an initial higher TNF-α, NO, and IL-10 response to pulmonary infection, PMΦ from the same strain showed a late and strong response mediated by H_2_O_2_ after intravenous *R*. *oryzae* infection. It is important to note that after infection via the intravenous route, the decrease in viable *R*. *oryzae* was inversely proportional to the release of H_2_O_2_ by PMΦ from a more resistant strain of mice. This observation suggests an important role of the response of PMΦ mediated by H_2_O_2_ in fungal control during disseminated mucormycosis. This result agrees with the data reported by Andrianaki et al. [33], who demonstrated the susceptibility of *A*. *fumigatus* and *R*. *oryzae* conidia to oxidative damage induced by H_2_O_2_. In this context, an important detail was that the BALB/c strain only showed a more efficient fungal clearance after 30 days of infection. We suggest that the adaptive immune response potentiated the activity of the macrophages evaluated in this study. However, more studies are needed to confirm this hypothesis.

Macrophages are the first immune cells that interact with invasive pathogens. Depending on their activity, macrophages can prime the adaptive immune response, which is far more aggressive and specific to pathogens [73]. Generally, high levels of ROS production by macrophages are linked to intracellular events, including activation of transcription factors such as nuclear factor-kappa B [74] and induction of mitogenesis [75]. In addition, *in vitro* and *in vivo* studies have already shown that *R*. *oryzae* could trigger a Th-17 response mediated by high levels of IL-23 in dendritic cells [76], as well as the association of high levels of IL-17 and IFN-γ with better *R*. *oryzae* elimination by immunocompetent BALB/c and C57BL/6 mice [30, 31]. Considering these prior and present findings, we hypothesize that adaptive immune responses developed by BALB/c mice are essential to enhance the fungicidal activity of PMΦ and efficiently kill *R*. *oryzae*.

The absence of additional experiments with live conidia to explore the direct role of reactive oxygen species in *R*. *oryzae* killing and to confirm the adaptive immune response related to a more efficient macrophage response to *R*. *oryzae*-Ag was the main limitation of the present study. Further studies should be performed to clarify the results observed here. It is also important to note that the conclusion obtained in this study is restricted to female mice and that future studies are needed to determine if this holds true in male mice.

In summary, our findings reveal that, independent of the female mouse strain, PMΦ are more reactive against *R*. *oryzae* in the first contact than AMΦ. In addition, increased PMΦ production of H_2_O_2_ at the end of the disseminated infection is accompanied by better fungal clearance in resistant (BALB/c) mice. Our findings provide new directions for understanding the parasite–host relationship in mucormycosis.

## Supporting information

S1 File (DOCX)

## References

[1] Severo LC, Oliveira FDM, Dreher R, Teixeira PZ, Porto NDS, Londero AT. Zygomycosis: A report of eleven cases and a review of the Brazilian literature. Rev Iberoam Micol. 2002;19: 52–56. 12716233

[2] Bitar D, Van Cauteren D, Lanternier F, Dannaoui E, Che D, Dromer F, et al. Increasing incidence of zygomycosis (mucormycosis), France, 1997–2006. Emerging Infect Dis. 2009;15: 1395–1401. doi: 10.3201/eid1509.090334 19788806 PMC2819884

[3] Kume H, Yamazaki T, Abe M, Tanuma H, Okudaira M, Okayasu I. Increase in aspergillosis and severe mycotic infection in patients with leukemia and MDS: comparison of the data from the Annual of the Pathological Autopsy Cases in Japan in 1989, 1993 and 1997. Pathol Int. 2003;53: 744–750. doi: 10.1046/j.1440-1827.2003.01548.x 14629297

[4] Neofytos D, Horn D, Anaissie E, Steinbach W, Olyaei A, Fishman J, et al. Epidemiology and outcome of invasive fungal infection in adult hematopoietic stem cell transplant recipients: analysis of Multicenter Prospective Antifungal Therapy (PATH) Alliance registry. Clin Infect Dis. 2009;48: 265–273. doi: 10.1086/595846 19115967

[5] Ribes JA, Vanover-Sams CL, Baker DJ. Zygomycetes in human disease. Clin Microbiol Rev. 2000;13: 236–301. doi: 10.1128/CMR.13.2.236 10756000 PMC100153

[6] Ibrahim AS, Spellberg B, Walsh TJ, Kontoyiannis DP. Pathogenesis of Mucormycosis. Clin Infect Dis. 2012;54: S16–S22. doi: 10.1093/cid/cir865 22247441 PMC3286196

[7] Roden MM, Zaoutis TE, Buchanan WL, Knudsen TA, Sarkisova TA, Schaufele RL, et al. Epidemiology and outcome of zygomycosis: a review of 929 reported cases. Clin Infect Dis. 2005;41: 634–653. doi: 10.1086/432579 16080086

[8] Spellberg B, Edwards J, Ibrahim A. Novel perspectives on mucormycosis: pathophysiology, presentation, and management. Clin Microbiol Rev. 2005;18: 556–569. doi: 10.1128/CMR.18.3.556-569.2005 16020690 PMC1195964

[9] Baddley JW, Thompson GR III, Chen SC-A, White PL, Johnson MD, Nguyen MH, et al. Coronavirus Disease 2019–Associated Invasive Fungal Infection. Open Forum Infectious Diseases. 2021;8: ofab510. doi: 10.1093/ofid/ofab510 34877364 PMC8643686

[10] Narayanan S, Chua JV, Baddley JW. COVID-19 associated Mucormycosis (CAM): risk factors and mechanisms of disease. Clin Infect Dis. 2021; ciab726. doi: 10.1093/cid/ciab726 34420052 PMC8499811

[11] Hussain S, Baxi H, Riad A, Klugarová J, Pokorná A, Slezáková S, et al. COVID-19-Associated Mucormycosis (CAM): An Updated Evidence Mapping. Int J Environ Res Public Health. 2021;18: 10340. doi: 10.3390/ijerph181910340 34639637 PMC8508302

[12] Rabagliati R, Rodríguez N, Núñez C, Huete A, Bravo S, Garcia P. COVID-19-Associated Mold Infection in Critically Ill Patients, Chile. Emerg Infect Dis. 2021;27: 1454–1456. doi: 10.3201/eid2705.204412 33760726 PMC8084475

[13] Patel A, Agarwal R, Rudramurthy SM, Shevkani M, Xess I, Sharma R, et al. Multicenter Epidemiologic Study of Coronavirus Disease–Associated Mucormycosis, India—Volume 27, Number 9—September 2021—Emerging Infectious Diseases journal—CDC. [cited 1 Feb 2022]. 10.3201/eid2709.210934 PMC8386807 34087089

[14] Hoenigl M, Seidel D, Carvalho A, Rudramurthy SM, Arastehfar A, Gangneux J-P, et al. The emergence of COVID-19 associated mucormycosis: a review of cases from 18 countries. The Lancet Microbe. 2022;0. doi: 10.1016/S2666-5247(21)00237-8 35098179 PMC8789240

[15] Bonates P, João GAP, Cruz KS, de S Ferreira M, Baía-da-Silva DC, de Farias MEL, et al. Fatal rhino-orbito-cerebral mucormycosis infection associated with diabetic ketoacidosis post-COVID-19. Rev Soc Bras Med Trop. 54: e0358–2021. doi: 10.1590/0037-8682-0358-2021 34259759 PMC8282253

[16] do Monte ES Junior, Santos MELD, Ribeiro IB, de O Luz G, Baba ER, Hirsch BS, et al. Rare and Fatal Gastrointestinal Mucormycosis (Zygomycosis) in a COVID-19 Patient: A Case Report. Clin Endosc. 2020;53: 746–749. doi: 10.5946/ce.2020.180 33207116 PMC7719411

[17] Pauli MA, de M Pereira L, Monteiro ML, de Camargo AR, Rabelo GD. Painful palatal lesion in a patient with COVID-19. Oral Surg Oral Med Oral Pathol Oral Radiol. 2021;131: 620–625. doi: 10.1016/j.oooo.2021.03.010 33867304 PMC8005255

[18] Nasir N, Farooqi J, Mahmood SF, Jabeen K. COVID-19 associated mucormycosis: a life-threatening complication in patients admitted with severe to critical COVID-19 from Pakistan. Clin Microbiol Infect. 2021;27: 1704–1707. doi: 10.1016/j.cmi.2021.07.038 34371205 PMC8349438

[19] Dulski TM. Notes from the Field: COVID-19–Associated Mucormycosis—Arkansas, July–September 2021. MMWR Morb Mortal Wkly Rep. 2021;70. doi: 10.15585/mmwr.mm7050a3 34914674 PMC8675658

[20] Mejía-Santos H. Notes from the Field: Mucormycosis Cases During the COVID-19 Pandemic—Honduras, May–September 2021. MMWR Morb Mortal Wkly Rep. 2021;70. doi: 10.15585/mmwr.mm7050a2 34914675 PMC8675660

[21] Cornely OA, Alastruey-Izquierdo A, Arenz D, Chen SCA, Dannaoui E, Hochhegger B, et al. Global guideline for the diagnosis and management of mucormycosis: an initiative of the European Confederation of Medical Mycology in cooperation with the Mycoses Study Group Education and Research Consortium. The Lancet Infectious Diseases. 2019;19: e405–e421. doi: 10.1016/S1473-3099(19)30312-3 31699664 PMC8559573

[22] Lelievre L, Garcia-Hermoso D, Abdoul H, Hivelin M, Chouaki T, Toubas D, et al. Posttraumatic mucormycosis: a nationwide study in France and review of the literature. Medicine (Baltimore). 2014;93: 395–404. doi: 10.1097/MD.0000000000000221 25500709 PMC4602436

[23] Neblett Fanfair R, Benedict K, Bos J, Bennett SD, Lo Y-C, Adebanjo T, et al. Necrotizing cutaneous mucormycosis after a tornado in Joplin, Missouri, in 2011. N Engl J Med. 2012;367: 2214–2225. doi: 10.1056/NEJMoa1204781 23215557

[24] Kontoyiannis DP, Lewis RE, Lortholary O, Lotholary O, Spellberg B, Petrikkos G, et al. Future directions in mucormycosis research. Clin Infect Dis. 2012;54 Suppl 1: S79–85. doi: 10.1093/cid/cir886 22247450 PMC3258101

[25] Skiada A, Lanternier F, Groll AH, Pagano L, Zimmerli S, Herbrecht R, et al. Diagnosis and treatment of mucormycosis in patients with hematological malignancies: guidelines from the 3rd European Conference on Infections in Leukemia (ECIL 3). Haematologica. 2013;98: 492–504. doi: 10.3324/haematol.2012.065110 22983580 PMC3659979

[26] Rodríguez MM, Pastor FJ, Calvo E, Salas V, Sutton DA, Guarro J. Correlation of in vitro activity, serum levels, and in vivo efficacy of posaconazole against Rhizopus microsporus in a murine disseminated infection. Antimicrob Agents Chemother. 2009;53: 5022–5025. doi: 10.1128/AAC.01026-09 19786601 PMC2786372

[27] Lewis RE, Albert ND, Liao G, Hou J, Prince RA, Kontoyiannis DP. Comparative pharmacodynamics of amphotericin B lipid complex and liposomal amphotericin B in a murine model of pulmonary mucormycosis. Antimicrob Agents Chemother. 2010;54: 1298–1304. doi: 10.1128/AAC.01222-09 20038620 PMC2826015

[28] Lewis RE, Liao G, Wang W, Prince RA, Kontoyiannis DP. Voriconazole pre-exposure selects for breakthrough mucormycosis in a mixed model of Aspergillus fumigatus-Rhizopus oryzae pulmonary infection. Virulence. 2011;2: 348–355. doi: 10.4161/viru.2.4.17074 21788730

[29] Lewis RE, Ben-Ami R, Best L, Albert N, Walsh TJ, Kontoyiannis DP. Tacrolimus enhances the potency of posaconazole against Rhizopus oryzae in vitro and in an experimental model of mucormycosis. J Infect Dis. 2013;207: 834–841. doi: 10.1093/infdis/jis767 23242544 PMC3563310

[30] dos Santos AR, Fraga-Silva TF, de F Almeida D, dos Santos RF, Finato AC, Amorim BC, et al. Rhizopus-host interplay of disseminated mucormycosis in immunocompetent mice. Future Microbiology. 2020 [cited 24 Jul 2020]. doi: 10.2217/fmb-2019-0246 32686962

[31] Dos Santos AR, Fraga-Silva TF, de Fátima Almeida-Donanzam D, Dos Santos RF, Finato AC, Soares CT, et al. IFN-γ Mediated Signaling Improves Fungal Clearance in Experimental Pulmonary Mucormycosis. Mycopathologia. 2021. doi: 10.1007/s11046-021-00598-2 34716549 PMC8555725

[32] Bao W, Jin L, Fu H, Shen Y, Lu G, Mei H, et al. Interleukin-22 Mediates Early Host Defense against Rhizomucor pusilluscan Pathogens. PLoS One. 2013;8. doi: 10.1371/journal.pone.0065065 23798999 PMC3684593

[33] Andrianaki AM, Kyrmizi I, Thanopoulou K, Baldin C, Drakos E, Soliman SSM, et al. Iron restriction inside macrophages regulates pulmonary host defense against Rhizopus species. Nature Communications. 2018;9: 3333. doi: 10.1038/s41467-018-05820-2 30127354 PMC6102248

[34] Zhang SX, Babady NE, Hanson KE, Harrington AT, Larkin PMK, Leal SM, et al. Recognition of Diagnostic Gaps for Laboratory Diagnosis of Fungal Diseases: Expert Opinion from the Fungal Diagnostics Laboratories Consortium (FDLC). J Clin Microbiol. 2021;59: e0178420. doi: 10.1128/JCM.01784-20 33504591 PMC8218742

[35] Venturini J, Golim MA, Alvares AM, Locachevic GA, Arruda OS, Arruda MSP. Morphofunctional evaluation of thymus in hyperglycemic-hypoinsulinemic mice during dermatophytic infection. FEMS Immunol Med Microbiol. 2011;62: 32–40. doi: 10.1111/j.1574-695X.2011.00784.x 21272093

[36] Russo M, Teixeira HC, Marcondes MC, Barbuto JA. Superoxide-independent hydrogen peroxide release by activated macrophages. Braz J Med Biol Res. 1989;22: 1271–1273. 2561630

[37] Fraga-Silva TFC, Marchetti CM, Mimura LAN, Locachevic GA, Golim MA, Venturini J, et al. Relationship among Short and Long Term of Hypoinsulinemia-Hyperglycemia, Dermatophytosis, and Immunobiology of Mononuclear Phagocytes. Mediators Inflamm. 2015;2015: 342345. doi: 10.1155/2015/342345 26538824 PMC4619976

[38] Green LC, Wagner DA, Glogowski J, Skipper PL, Wishnok JS, Tannenbaum SR. Analysis of nitrate, nitrite, and [15N]nitrate in biological fluids. Anal Biochem. 1982;126: 131–138. doi: 10.1016/0003-2697(82)90118-x 7181105

[39] Calich VL, Singer-Vermes LM, Siqueira AM, Burger E. Susceptibility and resistance of inbred mice to Paracoccidioides brasiliensis. Br J Exp Pathol. 1985;66: 585–594. 4063162 PMC2042050

[40] Radovanovic I, Mullick A, Gros P. Genetic control of susceptibility to infection with Candida albicans in mice. PLoS One. 2011;6: e18957. doi: 10.1371/journal.pone.0018957 21533108 PMC3080400

[41] Rhodes JC, Wicker LS, Urba WJ. Genetic Control of Susceptibility to Cryptococcus neoformans in Mice. Infect Immun. 1980;29: 494–499. doi: 10.1128/iai.29.2.494-499.1980 7216421 PMC551145

[42] Svirshchevskaya EV, Shevchenko MA, Huet D, Femenia F, Latgé J-P, Boireau P, et al. Susceptibility of mice to invasive aspergillosis correlates with delayed cell influx into the lungs. Int J Immunogenet. 2009;36: 289–299. doi: 10.1111/j.1744-313X.2009.00869.x 19744035

[43] Vilani-Moreno F, Fecchio D, de Mattos MC, Moscardi-Bacchi M, Defaveri J, Franco M. Study of pulmonary experimental paracoccidioidomycosis by analysis of bronchoalveolar lavage cells: resistant vs. susceptible mice. Mycopathologia. 1998;141: 79–91. 9750339 10.1023/a:1006987205848

[44] Hasenclever HF. Comparative pathogenicity of Candida albicans for mice and rabbits. J Bacteriol. 1959;78: 105–109. doi: 10.1128/jb.78.1.105-109.1959 13672915 PMC290489

[45] Maheshwari RK, Tandon RN, Feuillette AR, Mahouy G, Badillet G, Friedman RM. Interferon inhibits Aspergillus fumigatus growth in mice: an activity against an extracellular infection. J Interferon Res. 1988;8: 35–44. doi: 10.1089/jir.1988.8.35 2452848

[46] Waldorf AR, Ruderman N, Diamond RD. Specific susceptibility to mucormycosis in murine diabetes and bronchoalveolar macrophage defense against Rhizopus. J Clin Invest. 1984;74: 150–160. doi: 10.1172/JCI111395 6736246 PMC425195

[47] Bradley JR. TNF-mediated inflammatory disease. J Pathol. 2008;214: 149–160. doi: 10.1002/path.2287 18161752

[48] Belic S, Page L, Lazariotou M, Waaga-Gasser AM, Dragan M, Springer J, et al. Comparative Analysis of Inflammatory Cytokine Release and Alveolar Epithelial Barrier Invasion in a Transwell^®^ Bilayer Model of Mucormycosis. Front Microbiol. 2018;9: 3204. doi: 10.3389/fmicb.2018.03204 30671036 PMC6332705

[49] Wurster S, Thielen V, Weis P, Walther P, Elias J, Waaga-Gasser AM, et al. Mucorales spores induce a proinflammatory cytokine response in human mononuclear phagocytes and harbor no rodlet hydrophobins. Virulence. 2017;8: 1708–1718. doi: 10.1080/21505594.2017.1342920 28783439 PMC5810492

[50] Diamond RD, Clark RA. Damage to Aspergillus fumigatus and Rhizopus oryzae hyphae by oxidative and nonoxidative microbicidal products of human neutrophils in vitro. Infect Immun. 1982;38: 487–495. doi: 10.1128/iai.38.2.487-495.1982 6292103 PMC347765

[51] Diamond RD, Haudenschild CC, Erickson NF. Monocyte-mediated damage to Rhizopus oryzae hyphae in vitro. Infect Immun. 1982;38: 292–297. doi: 10.1128/iai.38.1.292-297.1982 7141693 PMC347731

[52] de CR Gonçalves R, Kitagawa RR, Raddi MSG, Carlos IZ, Pombeiro-Sponchiado SR. Inhibition of nitric oxide and tumour necrosis factor-α production in peritoneal macrophages by Aspergillus nidulans melanin. Biol Pharm Bull. 2013;36: 1915–1920. 24432378 10.1248/bpb.b13-00445

[53] Heinekamp T, Thywissen A, Macheleidt J, Keller S, Valiante V, Brakhage AA. Aspergillus fumigatus melanins: interference with the host endocytosis pathway and impact on virulence. Front Microbiol. 2013;3. doi: 10.3389/fmicb.2012.00440 23346079 PMC3548413

[54] Guth AM, Janssen WJ, Bosio CM, Crouch EC, Henson PM, Dow SW. Lung environment determines unique phenotype of alveolar macrophages. Am J Physiol Lung Cell Mol Physiol. 2009;296: L936–946. doi: 10.1152/ajplung.90625.2008 19304907 PMC2692811

[55] Thepen T, Van Rooijen N, Kraal G. Alveolar macrophage elimination in vivo is associated with an increase in pulmonary immune response in mice. J Exp Med. 1989;170: 499–509. doi: 10.1084/jem.170.2.499 2526847 PMC2189410

[56] Leopold Wager CM, Wormley FL. Classical versus alternative macrophage activation: the Ying and the Yang in host defense against pulmonary fungal infections. Mucosal Immunol. 2014;7: 1023–1035. doi: 10.1038/mi.2014.65 25073676

[57] Salim T, Sershen CL, May EE. Investigating the Role of TNF-α and IFN-γ Activation on the Dynamics of iNOS Gene Expression in LPS Stimulated Macrophages. PLoS One. 2016;11: e0153289. doi: 10.1371/journal.pone.0153289 27276061 PMC4898755

[58] Moreira AP, Dias-Melicio LA, Peraçoli MTS, Calvi SA, Victoriano de Campos Soares AM. Killing of Paracoccidioides brasiliensis yeast cells by IFN-gamma and TNF-alpha activated murine peritoneal macrophages: evidence of H(2)O (2) and NO effector mechanisms. Mycopathologia. 2008;166: 17–23. doi: 10.1007/s11046-007-9046-3 18496766

[59] Kawakami K, Qureshi MH, Koguchi Y, Zhang T, Okamura H, Kurimoto M, et al. Role of TNF-alpha in the induction of fungicidal activity of mouse peritoneal exudate cells against Cryptococcus neoformans by IL-12 and IL-18. Cell Immunol. 1999;193: 9–16. doi: 10.1006/cimm.1999.1460 10202108

[60] Roilides E, Dimitriadou-Georgiadou A, Sein T, Kadiltsoglou I, Walsh TJ. Tumor necrosis factor alpha enhances antifungal activities of polymorphonuclear and mononuclear phagocytes against Aspergillus fumigatus. Infect Immun. 1998;66: 5999–6003. doi: 10.1128/IAI.66.12.5999-6003.1998 9826384 PMC108760

[61] Mehrad B, Strieter RM, Standiford TJ. Role of TNF-alpha in pulmonary host defense in murine invasive aspergillosis. J Immunol. 1999;162: 1633–1640. 9973423

[62] Keijzer A, van der Valk P, Ossenkoppele GJ, van de Loosdrecht A. Mucormycosis in a patient with low risk myelodysplasia treated with anti-TNF-alpha. Haematologica. 2006;91: ECR51–ECR51. 17194657

[63] Singh P, Taylor SF, Murali R, Gomes LJ, Kanthan GL, Maloof AJ. Disseminated mucormycosis and orbital ischaemia in combination immunosuppression with a tumour necrosis factor alpha inhibitor. Clinical & Experimental Ophthalmology. 2007;35: 275–280. doi: 10.1111/j.1442-9071.2007.01458.x 17430516

[64] Gonzalez A, de Gregori W, Velez D, Restrepo A, Cano LE. Nitric oxide participation in the fungicidal mechanism of gamma interferon-activated murine macrophages against Paracoccidioides brasiliensis conidia. Infect Immun. 2000;68: 2546–2552. doi: 10.1128/IAI.68.5.2546-2552.2000 10768942 PMC97457

[65] Bocca AL, Hayashi EE, Pinheiro AG, Furlanetto AB, Campanelli AP, Cunha FQ, et al. Treatment of Paracoccidioides brasiliensis-infected mice with a nitric oxide inhibitor prevents the failure of cell-mediated immune response. J Immunol. 1998;161: 3056–3063. 9743371

[66] Nascimento FRF, Calich VLG, Rodríguez D, Russo M. Dual role for nitric oxide in paracoccidioidomycosis: essential for resistance, but overproduction associated with susceptibility. J Immunol. 2002;168: 4593–4600. doi: 10.4049/jimmunol.168.9.4593 11971007

[67] Couper KN, Blount DG, Riley EM. IL-10: The Master Regulator of Immunity to Infection. The Journal of Immunology. 2008;180: 5771–5777. doi: 10.4049/jimmunol.180.9.5771 18424693

[68] Roilides E, Dimitriadou A, Kadiltsoglou I, Sein T, Karpouzas J, Pizzo PA, et al. IL-10 exerts suppressive and enhancing effects on antifungal activity of mononuclear phagocytes against Aspergillus fumigatus. The Journal of Immunology. 1997;158: 322–329. 8977206

[69] Grünig G, Corry DB, Leach MW, Seymour BW, Kurup VP, Rennick DM. Interleukin-10 is a natural suppressor of cytokine production and inflammation in a murine model of allergic bronchopulmonary aspergillosis. J Exp Med. 1997;185: 1089–1099. doi: 10.1084/jem.185.6.1089 9091582 PMC2196229

[70] Dinarello CA. Biologic basis for interleukin-1 in disease. Blood. 1996;87: 2095–2147. 8630372

[71] Netea MG, Sutmuller R, Hermann C, der Graaf CAAV, der Meer JWMV, van Krieken JH, et al. Toll-Like Receptor 2 Suppresses Immunity against Candida albicans through Induction of IL-10 and Regulatory T Cells. The Journal of Immunology. 2004;172: 3712–3718. doi: 10.4049/jimmunol.172.6.3712 15004175

[72] Potenza L, Vallerini D, Barozzi P, Riva G, Forghieri F, Zanetti E, et al. Mucorales-specific T cells emerge in the course of invasive mucormycosis and may be used as a surrogate diagnostic marker in high-risk patients. Blood. 2011;118: 5416–5419. doi: 10.1182/blood-2011-07-366526 21931119

[73] Ghuman H, Voelz K. Innate and Adaptive Immunity to Mucorales. J Fungi (Basel). 2017;3. doi: 10.3390/jof3030048 29371565 PMC5715954

[74] Sun Y, Oberley LW. Redox regulation of transcriptional activators. Free Radical Biology and Medicine. 1996;21: 335–348. doi: 10.1016/0891-5849(96)00109-8 8855444

[75] Joneson T, Bar-Sagi D. A Rac1 Effector Site Controlling Mitogenesis through Superoxide Production. J Biol Chem. 1998;273: 17991–17994. doi: 10.1074/jbc.273.29.17991 9660749

[76] Chamilos G, Ganguly D, Lande R, Gregorio J, Meller S, Goldman WE, et al. Generation of IL-23 producing dendritic cells (DCs) by airborne fungi regulates fungal pathogenicity via the induction of T(H)-17 responses. PLoS ONE. 2010;5: e12955. doi: 10.1371/journal.pone.0012955 20886035 PMC2944889

