# Supplementary material for: Is the production of reactive oxygen and nitrogen species by macrophages associated with better infectious control in female mice with experimentally disseminated and pulmonary mucormycosis?
Source: PLoS One. 2022 Dec 15;17(12):e0270071. doi: 10.1371/journal.pone.0270071 (PMC9754262; doi:10.1371/journal.pone.0270071)
Supplement: S1 File — (DOCX) [file pone.0270071.s001.docx]

**Supporting Information**

**Table 1. NO, H_2_O_2_, TNF-α, and IL-10 levels in cell-free supernatants peritoneal macrophages from naïve Swiss mice co-cultured or not with heat-killed spores of *R. oryzae.***

| **Samples** | NO Ø | NO Rhi-Ag | H_2_O_2_ Ø | H_2_O_2_ Rhi-Ag | TNF-α Ø | TNF-α Rhi-Ag | IL-10 Ø | IL-10 Rhi-Ag |
| --- | --- | --- | --- | --- | --- | --- | --- | --- |
| **A1** | 20.63609 | 3.81589 | 3.741403 | 2.078161 | 628.6676 | 3611.306 | 1910.56 | 940.4087 |
| **A2** | 24.02371 | 4.664289 | 0.516236 | 3.064063 | 827.3705 | 4447.008 | 932.1621 | 1346.504 |
| **A3** | 23.66716 | 7.063385 | 2.285034 | 1.653691 | 691.5081 | 3375.62 | 2228.021 | 725.4886 |
| **A4** | 18.67628 | 3.81589 | 0.880632 | 2.078161 | 862.2035 | 4595.957 | 2194.244 | 725.4886 |
| **A5** | 25.62755 | 6.890964 | 1.363531 | 1.595438 | 860.7333 | 4515.906 | 2153.39 | 4884.354 |

*NO: Nitric oxide; H2O2: Hydrogen peroxide; Rhi-Ag: heat-killed spores of *R. oryzae*. NO and H_2_O_2_ values are expressed in nM/mL. IL-10 and TNF-α values are expressed in pg/mL.

**Table 2. NO, H_2_O_2_, TNF-α, and IL-10 levels in cell-free supernatants peritoneal macrophages from naïve BALB/c mice co-cultured or not with heat-killed spores of *R. oryzae*.**

| **Samples** | NO Ø | NO Rhi-Ag | H_2_O_2_ Ø | H_2_O_2_ Rhi-Ag | TNF-α Ø | TNF-α Rhi-Ag | IL-10 Ø | IL-10 Rhi-Ag |
| --- | --- | --- | --- | --- | --- | --- | --- | --- |
| **A1** | 36.43096 | 8.447997 | 1.5702 | 2.084821 | 628.6676 | 3611.306 | 1735.115 | 4397.959 |
| **A2** | 40.80918 | 7.408679 | 1.054508 | 3.589076 | 827.3705 | 4447.008 | 1811.164 | 4877.551 |
| **A3** | 23.48887 | 8.447997 | 1.420985 | 4.778131 | 691.5081 | 3375.62 | 2240.916 | 4017.007 |
| **A4** | 26.69596 | 6.718695 | 1.400573 | 2.666285 | 862.2035 | 4595.957 | 2224.871 | 4724.49 |
| **A5** | 30.42742 | 5.004958 |  | 3.746838 | 860.7333 | 4515.906 | 863.5761 | 4952.381 |

*NO: Nitric oxide; H2O2: Hydrogen peroxide; Rhi-Ag: heat-killed spores of *R. oryzae*. NO and H_2_O_2_ values are expressed in nM/mL. IL-10 and TNF-α values are expressed in pg/mL.

**Table 3. NO, H_2_O_2_, TNF-α, and IL-10 levels in cell-free supernatants alveolar macrophages from naïve Swiss mice co-cultured or not with heat-killed spores of *R. oryzae*.**

| **Samples** | NO Ø | NO Rhi-Ag | H_2_O_2_ Ø | H_2_O_2_ Rhi-Ag | TNF-α Ø | TNF-α Rhi-Ag | IL-10 Ø | IL-10 Rhi-Ag |
| --- | --- | --- | --- | --- | --- | --- | --- | --- |
| **A1** | 4.3885 | 2.359803 | 0.381703 | 0.513754 | 338.9117 | 309.1423 | 0 | 0 |
| **A2** | 2.359803 | 2.359803 | 0.56846 | 0.6784 | 997.8856 | 947.595 | 0 | 0 |
| **A3** | 1.858704 | 2.359803 | 0.507801 | 0.543883 | 542.7246 | 560.5625 | 0 | 0 |
| **A4** | 2.192485 | 2.695276 | 0.51973 | 0.495965 | 1007.909 | 834.5521 | 0 | 0 |
| **A5** | 2.527401 | 2.192485 | 0.543883 | 0.432325 | 575.8957 | 420.181 | 0 | 0 |

*NO: Nitric oxide; H2O2: Hydrogen peroxide; Rhi-Ag: heat-killed spores of *R. oryzae*. NO and H_2_O_2_ values are expressed in nM/mL. IL-10 and TNF-α values are expressed in pg/mL.

**Table 4. NO, H_2_O_2_, TNF-α, and IL-10 levels in cell-free supernatants alveolar macrophages from naïve BALB/c mice co-cultured or not with heat-killed spores of *R. oryzae*.**

| **Samples** | NO Ø | NO Rhi-Ag | H_2_O_2_ Ø | H_2_O_2_ Rhi-Ag | TNF-α Ø | TNF-α Rhi-Ag | IL-10 Ø | IL-10 Rhi-Ag |
| --- | --- | --- | --- | --- | --- | --- | --- | --- |
| **A1** | 3.200527 | 2.527401 | 0.543883 | 0.685177 | 955.5455 | 1345.197 | 0 | 0 |
| **A2** | 3.708147 | 3.877864 | 0.671661 | 0.572599 | 811.7009 | 1052.537 | 0 | 0 |
| **A3** | 2.192485 | 3.708147 | 0.593493 | 0.614728 | 542.7246 | 1104.824 | 0 | 0 |
| **A4** | 2.527401 | 6.625077 | 0.46097 | 2.445785 |  | 1248.318 | 0 | 0 |
| **A5** |  | 7.669075 |  |  |  |  | 0 | 0 |

*NO: Nitric oxide; H2O2: Hydrogen peroxide; Rhi-Ag: heat-killed spores of *R. oryzae*. NO and H_2_O_2_ values are expressed in nM/mL. IL-10 and TNF-α values are expressed in pg/mL.

**Table 5. Viable fungal recovery (%) in Swiss mice after 7 and 30 days of intravenous infection of with 3 × 10^4^ spores of *R. oryzae*.**

| **Samples** | **Brain 7d** | **Brain 30d** | **Kidney 7d** | **Kidney 30d** | **Liver 7d** | **Liver 30d** | **Lung 7d** | **Lung 30d** | **Spleen 7d** | **Spleen 30d** |
| --- | --- | --- | --- | --- | --- | --- | --- | --- | --- | --- |
| **A1** | 25 | 0 | 50 | 40 | 100 | 20 | 75 | 20 | 100 | 100 |
| **A2** | 25 | 20 | 45 | 0 | 100 | 50 | 75 | 40 | 100 | 100 |
| **A3** | 0 | 0 | 20 | 20 | 40 | 20 | 50 | 20 | 100 | 33 |
| **A4** | 0 | 0 | 20 | 20 | 100 | 100 | 80 | 20 | 100 | 100 |
| **A5** | 50 | 20 | 50 | 40 | 100 | 20 | 75 | 20 | 99 | 100 |

* values are expressed in % of positive fragments.

**Table 6. NO, H_2_O_2_, TNF-α, and IL-10 levels in cell-free supernatants of peritoneal macrophages from Swiss mice evaluated after 7 and 30 days of intravenous infection of with 3 × 10^4^ spores of *R. oryzae*.**

| **Samples** | NO 7d | NO 30d | H_2_O_2_ 7d | H_2_O_2_ 30d | TNF-α 7d | TNF-α 30d | IL-10 7d | IL-10 30d |
| --- | --- | --- | --- | --- | --- | --- | --- | --- |
| **A1** | 38.34611 | 3.872678 | 0.97931 | 0.550488 | 79336.91 | 123.5107 | 2035.016 | 94.54701 |
| **A2** | 18.761 | 1.306354 | 1.002748 | 0.764499 | 11684.85 | 4329.597 | 209.1897 | 250.4995 |
| **A3** | 60.34457 | 2.269835 | 0.725933 | 0.824005 | 16940.25 | 5186.76 | 2192.159 | 201.4081 |
| **A4** | 38.34611 | 1.480585 | 0.531542 | 0.784188 | 16079.23 | 760.9541 | 125.4934 | 95.39172 |
| **A5** | 18.761 | 7.258397 | 1.223097 | 0.706274 | 43419.72 | 844.7434 |  | 95.39172 |

*NO: Nitric oxide; H2O2: Hydrogen peroxide. NO and H2O2 values are expressed in nM/mL. IL-10 and TNF-α values are expressed in pg/mL. The peritoneal macrophages were co-cultivated with heat-killed spores of *R. oryzae.*

**Table 7. Viable fungal recovery (%) in BALB/c mice after 7 and 30 days of intravenous infection of with 3 × 10^4^ spores of *R. oryzae*.**

| **Samples** | **Brain 7d** | **Brain 30d** | **Kidney 7d** | **Kidney 30d** | **Liver 7d** | **Liver 30d** | **Lung 7d** | **Lung 30d** | **Spleen 7d** | **Spleen 30d** |
| --- | --- | --- | --- | --- | --- | --- | --- | --- | --- | --- |
| **A1** | 100 | 0 | 50 | 20 | 100 | 60 | 80 | 20 | 100 | 1 |
| **A2** | 20 | 0 | 60 | 0 | 100 | 20 | 60 | 0 | 100 | 0 |
| **A3** | 0 | 0 | 40 | 0 | 100 | 20 | 80 | 20 | 100 | 0 |
| **A4** | 25 | 0.1 | 24 | 20 | 80 | 20 | 25 | 0 | 99 | 0 |

* values are expressed in % of positive fragments.

**Table 8. NO, H_2_O_2_, TNF-α, and IL-10 levels in cell-free supernatants of peritoneal macrophages from BALB/c mice evaluated after 7 and 30 days of intravenous infection of with 3 × 10^4^ spores of *R. oryzae*.**

| **Samples** | NO 7d | NO 30d | H_2_O_2_ 7d | H_2_O_2_ 30d | TNF-α 7d | TNF-α 30d | IL-10 7d | IL-10 30d |
| --- | --- | --- | --- | --- | --- | --- | --- | --- |
| **A1** | 5.846583 | 4.323356 | 1.332744 | 7.583108 | 570.9769 | 659.8631 | 1323.356 | 255.8179 |
| **A2** | 9.886489 | 2.358045 | 0.51352 | 7.757964 | 3259.196 | 564.122 | 573.9112 | 252.6127 |
| **A3** | 6.716316 | 4.323356 | 0.346438 | 8.013592 | 2134.413 | 459.3477 | 672.0379 | 112.4358 |
| **A4** | 9.886489 | 2.97832 | 1.041292 |  | 1968.975 | 529.6094 | 1810.673 | 631.4404 |
| **A5** | 4.634836 | 1.830321 | 0.746742 |  | 2134.413 | 558.6872 | 602.1668 | 112.4358 |

*NO: Nitric oxide; H2O2: Hydrogen peroxide. NO and H2O2 values are expressed in nM/mL. IL-10 and TNF-α values are expressed in pg/mL. The peritoneal macrophages were co-cultivated with heat-killed spores of *R. oryzae.*

**Table 9. Viable fungal recovery (%) in Swiss mice after 7 and 30 days of intratracheal inoculation of 2 ×10^6^ spores of *R. oryzae*.**

| **Samples** | Brain 7d | Brain 30d | Kidney 7d | Kidney 30d | Liver 7d | Liver 30d | Lung 7d | Lung 30d | Spleen 7d | Spleen 30d |
| --- | --- | --- | --- | --- | --- | --- | --- | --- | --- | --- |
| **A1** | 0 | 0 | 0 | 0 | 60 | 0 | 100 | 0 | 66 | 20 |
| **A2** | 0 | 0 | 0 | 75 | 60 | 0 | 100 | 0 | 66 | 100 |
| **A3** | 100 | 0 | 100 | 0 | 20 | 0 | 100 | 100 | 100 | 0 |
| **A4** | 0 | 0 | 20 | 0 | 20 | 20 | 100 | 60 | 20 | 20 |
| **A5** | 80 | 20 | 0 | 0 | 100 | 40 | 80 | 60 | 50 | 20 |

* values are expressed in % of positive fragments.

**Table 10. NO, H_2_O_2_, TNF-α, and IL-10 levels in cell-free supernatants of alveolar macrophages from Swiss mice evaluated after 7 and 30 days of intratracheal with 2 ×10^6^ spores of *R. oryzae*.**

| **Samples** | NO 7d | NO 30d | H_2_O_2_ 7d | H_2_O_2_ 30d | TNF-α 7d | TNF-α 30d | IL-10 7d | IL-10 30d |
| --- | --- | --- | --- | --- | --- | --- | --- | --- |
| **A1** | 5.31226 | 3.677163 | 3.670377 | 0.289349 | 697.6268 | 525.1564 | 0 | 0 |
| **A2** | 5.31226 | 2.241673 | 2.574011 | 0 | 828.9073 | 1121.615 | 0 | 0 |
| **A3** | 4.402666 | 15.81406 | 2.218198 | 0 | 320.9283 | 857.8493 | 0 | 0 |
| **A4** | 3.665784 | 1.178494 | 2.355915 |  | 311.8074 | 2187.757 | 0 | 0 |
| **A5** |  | 2.956992 |  |  |  |  | 0 | 0 |

*NO: Nitric oxide; H2O2: Hydrogen peroxide. NO and H2O2 values are expressed in nM/mL. IL-10 and TNF-α values are expressed in pg/mL. The peritoneal macrophages were co-cultivated with heat-killed spores of *R. oryzae.*

**Table 11. Viable fungal recovery (%) in BALB/c mice after 7 and 30 days of intratracheal inoculation of 2 ×10^6^ spores of *R. oryzae*.**

| **Samples** | Brain 7d | Brain 30d | Kidney 7d | Kidney 30d | Liver 7d | Liver 30d | Lung 7d | Lung 30d | Spleen 7d | Spleen 30d |
| --- | --- | --- | --- | --- | --- | --- | --- | --- | --- | --- |
| **A1** | 100 | 0 | 20 | 0 | 100 | 0 | 100 | 50 | 100 | 0 |
| **A2** | 80 | 0 | 100 | 0 | 100 | 20 | 100 | 40 | 100 | 0 |
| **A3** | 100 | 0 | 20 | 0 | 40 | 0 | 100 | 20 | 100 | 0 |
| **A4** | 40 | 0 | 80 | 0 | 40 | 0 | 100 | 0 | 60 | 0 |
| **A5** | 40 | 0.1 | 100 | 0.1 | 60 | 0 | 100.1 | 0 | 40 | 0.1 |

* values are expressed in % of positive fragments.

**Table 12. NO, H_2_O_2_, TNF-α, and IL-10 levels in cell-free supernatants of alveolar macrophages from BALB/c mice evaluated after 7 and 30 days of intratracheal inoculation of 2 ×10^6^ spores of *R. oryzae.***

| **Samples** | NO 7d | NO 30d | H_2_O_2_ 7d | H_2_O_2_ 30d | TNF-α 7d | TNF-α 30d | IL-10 7d | IL-10 30d |
| --- | --- | --- | --- | --- | --- | --- | --- | --- |
| **A1** | 14.85866 | 2.42003 | 0.448042 | 0 | 1497.853 | 1130.699 | 81.43091 | 0 |
| **A2** | 19.06384 | 2.42003 | 0.307868 | 0 | 2379.794 | 492.4006 | 96.23304 | 0 |
| **A3** | 4.767998 | 2.598706 | 0.349177 | 0.490311 | 2302.511 | 240.92 | 78.6974 | 0 |
| **A4** | 23.72349 | 6.415743 | 0.187753 |  | 1939.149 | 1945.727 | 0 | 0 |
| **A5** |  | 4.401872 | 0 |  | 1834.764 |  | 0 | 0 |
| **A6** |  |  | 0 |  |  |  |  |  |

*NO: Nitric oxide; H2O2: Hydrogen peroxide. NO and H2O2 values are expressed in nM/mL. IL-10 and TNF-α values are expressed in pg/mL. The peritoneal macrophages were co-cultivated with heat-killed spores of *R. oryzae*.
